# Neuroendocrine and behavioral response to social rupture and repair in preschoolers with autism spectrum disorders interacting with mother and father

**DOI:** 10.1186/s13229-015-0007-2

**Published:** 2015-03-06

**Authors:** Sharon Ostfeld-Etzion, Ofer Golan, Yael Hirschler-Guttenberg, Orna Zagoory-Sharon, Ruth Feldman

**Affiliations:** Department of Psychology, Bar-Ilan University, Ramat-Gan, Israel; The Association for Children at Risk, Tel-Aviv, Israel; The Gonda Brain Research Center, Bar-Ilan University, Ramat-Gan, Israel

**Keywords:** Preschoolers, Mothering, Fathering, HPA, Cortisol, Emotion regulation, Still face

## Abstract

**Background:**

Preschoolers with autism spectrum disorder (ASD) exhibit difficulties in handling social stress and utilizing efficient emotion regulation (ER) strategies to manage high arousal. While researchers called to assess ER in ASD, few studies utilized direct observations. We tested children’s behavioral and cortisol response to maternal and paternal unavailability and hypothesized that children with ASD will employ less complex ER strategies and their parents would show increased regulation facilitation effort to accommodate their child’s difficulties.

**Methods:**

Forty preschoolers with ASD were matched with 40 typically developing (TD) preschoolers. Children were seen twice for identical battery with mother or father in the face-to-face-still-face paradigm, a three-episode paradigm where parent-child play (free play (FP)) is interrupted by elimination of communication (still face (SF)) followed by resuming play (reunion (RE)). Micro-coding of parent and child’s social behavior and ER strategies was conducted. Parent and child’s cortisol was assessed at baseline, following stress, and at recovery.

**Results:**

Children with ASD exhibited the typical SF effect, indexed by an increase in negative affect and decrease in positive communications, but employed more simple regulatory behavior (self-soothing, proximity-seeking) and less complex strategies involving attention redirection and substitutive play. Their parents used more regulation-facilitation behavior, both simple and complex. All children showed initial cortisol response to novelty, which declined over time. However, maternal presence suppressed initial cortisol response in children with ASD.

**Conclusions:**

Children with ASD form typical expectations of parental availability and their parents increase effort to help repair social rupture. Among children with ASD, maternal presence and regulation facilitation provide social buffering for the child’s HPA stress response in a manner similar to mammalian neonates. Results highlight the importance of assessing ER by combining direct observations and physiological measures and including fathers in empirical studies and intervention efforts for children with ASD during sensitive periods for social growth.

**Electronic supplementary material:**

The online version of this article (doi:10.1186/s13229-015-0007-2) contains supplementary material, which is available to authorized users.

## Background

Children enter the social world through the careful adaptation of an attuned caregiver who introduces the rules of social exchange in repeatedly experienced social encounters [1,2]. During such social moments, infants learn to jointly build social interactions from the nonverbal patterns of self and others and practice the conventions of social partnership [3]. The parent’s moment-by-moment integration of the infant into the social unit - an experience variously termed ‘synchrony’, ‘mutual regulation’, or ‘attunement’ - provides critical inputs during a sensitive period for the development of the social brain, affords external-regulatory support for maturation of the stress response, and bears long-term impact on the child’s cognitive, social, and emotional growth [3-5]. Participation in rule-governed social exchange enables children to develop social expectations and serves as the basis for the child’s understanding others’ intentions, desires, and goals via their social action, that is, ‘theory-of-mind’ [6]. In typically developing (TD) children, social expectations are formed during parent-infant face-to-face interactions between the age of 3 and 6 months, the most social period of human life before play becomes focused on object exploration and when the infant’s active engagement with the world occurs mainly through the coordination of visuo-affective social signals [2-5].

One paradigm designed to test infants’ internalization of social expectations is the face-to-face still-face (FTFSF) paradigm. In this paradigm, the parent engages with the child for 3 min, refrains from social communication, and maintains a still face for 2 to 3 min, and resumes typical play for additional 2 min. Extant research using the FTFSF paradigm, mainly with 3- to 6-month-old infants, has shown that by 3 months infants have already formed social expectations of parental availability, decrease positive behavior, and increase negative emotionality and withdrawal during parental still face, and carryover effects of the social disruption is observed during the ‘reparation’ phase at reunion [7,8]. Such moments of social rupture and repair, typical of human communication, enable the display of the infant’s regulatory skills when facing a social stressor [9]. Similar to attachment security, which is measured by the infant’s regulated response to moments of maternal separation and return, the infant’s ability to handle parental unavailability using age-appropriate tactics may provide an index of the child’s regulatory competencies and manifests the parent’s capacity to facilitate emotion-regulatory abilities in the child [10].

Although most studies examined the SF effect with mothers, the few studies comparing infants’ response to parental SF in the presence of mother versus father found little differences and showed that children display the SF effect to both parents and their emotion regulation (ER) behavior during moments of parental unavailability is associated with both mothers’ and fathers’ general interactive sensitivity [8]. Very little research utilized the FTFSF paradigm beyond the first months of life to test preschoolers’ regulatory strategies during parental unavailability. One study of preschoolers [11] found that during maternal still face children used both *putative regulatory behavior* - behaviors whose only goal is self-regulation, such as self-soothing, repetitive self-talk, or proximity-seeking, and *complex regulatory behaviors* - behaviors that are not inherently regulatory but are used for regulation during moments of stress, such as substitutive play or attention redirection. Children with more advanced social skills and those receiving more attuned parenting displayed greater use of complex regulatory tactics, indicating that the strategies preschoolers use to regulate social rupture and repair may index the degree of social maturity. However, to date, no study examined preschoolers’ response to parental unavailability in the presence of both mother and father.

Autism spectrum disorder (ASD) is a neurodevelopmental condition marked by social-communication deficits and restricted, repetitive behaviors. Abilities tapped by the FTFSF paradigm, such as forming social expectations, internalizing social rules, understanding mental states, and regulating moments of emotional distress, are disrupted in children with ASD [12-14], suggesting that the paradigm may tap the specific social-regulatory difficulties in ASD. Generally, studies of ER skills as measured by direct observations of behavior in emotion-eliciting contexts are rare in ASD and authors have underscored the need for much further research on the specific ER behaviors children with ASD use to regulate moments of social stress [15]. The few existing studies examined child ER in situations that elicit anger or frustration [16,17] and showed that preschoolers with ASD employ more simple and physical regulatory tactics, such as self-soothing or proximity-seeking, which are typically observed in infants and toddlers [11], and less complex strategies that rely on cognitive or attentive processes, such as attention diversion or substitutive play. Difficulties in affective sharing [18], limited understanding of emotional messages [12], reduced gaze synchrony [19], and immature theory-of-mind abilities [20], characteristic of preschoolers with ASD, further complicate their ability to regulate social stress. Interestingly, Field and colleagues [21] used variation of the FTFSF paradigm in preschoolers with ASD during interactions with strangers. Following stranger imitation, but not before, children initiated social contact during the stranger’s SF, but the response of children with ASD to their parents’ SF has not yet been tested.

Very little research examined ER behavior in children with ASD in the presence of their mother and none in the presence of their father. Mothers of children with ASD were found to use simple regulation-facilitation tactics, such as physical proximity, to assist the regulation of stress [22]. Overall, fathering in ASD received extremely little attention with nearly no study utilizing direct observations. Children with ASD initiated less joint attention and social gaze with their fathers and exhibited more self-stimulation compared to TD children [23], and fathers were found to be less active in promoting social engagement than mothers [17,24,25]. Describing the unique ER behavior children with ASD use with their mother and father is important in order to tease apart regulatory strategies associated with the disorder from those that are expressed only in a specific parent-child context.

Social rupture and repair, as measured by the FTFSF paradigm, elicit neuroendocrine stress response from infants. Infants show cortisol increase following maternal SF [26], and infants of more sensitive mothers exhibit more regulated cortisol response [27]. Cortisol, the end product of the hypothalamic-pituitary-adrenal (HPA) axis, increases in response to social stress [28-30] in both mammals [31,32] and humans [33,34]. Research in humans and other mammals has emphasized the importance of the mother’s proximity and ongoing social cues for the ‘social buffering’ of the infant’s stress response, that is, the suppression of infant HPA reactivity by the mother’s presence [35]. Animal studies have demonstrated that during the first days of life, infant rodents show a ‘stress hypo-responsive period’, a time when the stress response is not yet active. This is followed by a period of ‘social buffering’ - a period when the infant’s HPA system is active but is suppressed by the mother’s presence. Following this transition period, the HPA system becomes fully active, enabling the infant to meet the world and its dangers [36]. Studies have also shown that the ‘social buffering’ effect disappears in cases of maternal deprivation and can be reinstated by artificial licking.

Despite mixed results on cortisol patterns in children with ASD, no systematic differences were found between TD and ASD children in basal levels [37], suggesting that the HPA system may function in a similar manner in the two groups. Greater diurnal cortisol variability, but not production, was found in preschoolers with ASD [38]. Three-to-nine year olds with ASD who showed more repetitive behavior exhibited lower diurnal cortisol, indicating that repetitive behavior may serve an anti-stress function for these children [39]. Cortisol response to social stressors showed no differences between ASD and TD children [40], and no differences were found following a sensory challenge [41]. However, cortisol increase was observed when children were required to play with an unfamiliar peer [42], suggesting that strangers may elicit a stress response.

In light of the above, the current study adapted the FTFSF paradigm to study social expectations and ER behavior in preschoolers with ASD, compared to matched TD children, during interactions with mother and father (separately). We conjectured that using a paradigm that taps the roots of social behavior during the first period of human social life, when the social difficulties of children who later develop autism are not yet manifest, may provide new insights on the social-regulatory difficulties in this group. Assessing ER behavior with both mother and father and in relation to both micro-level behavioral patterns and neuroendocrine stress response was thought to provide a novel viewpoint not previously addressed. Furthermore, authors have noted [15] that whereas much research is needed to understand the development of ER in children with ASD, regulatory behaviors may have different goals or meaning in this group and strategies that successfully regulate negative arousal in TD children may be ineffective in children with ASD. As such, the FTFSF paradigm, where it is possible to test whether specific ER behaviors were indeed helpful to children in repairing social engagement at the next step, may provide a unique window to study ER processes in this group.

Four hypotheses were proposed. First, in light of research showing similar distributions of secure attachment in children with high-functioning ASD and TD children [43], we expected that children with ASD would exhibit a similar SF effect to that of TD children, expressed by increase in negative and decrease in positive behavior during moments of both maternal and paternal unavailability. Second, we expected that children with ASD would use more simple regulatory strategies during social rupture in comparison with TD children, whereas the latter would employ more complex regulatory behaviors. Third, we hypothesized that mothers and fathers of children with ASD would exhibit more regulation-facilitation behavior following social rupture, to help their children get back into the social-communicative unit. Finally, we expected that social stress would elicit cortisol response from all children and that parental presence would function to regulate the child’s HPA reactivity, possibly in different ways in each group and with each parent.

## Methods

### Participants

The sample included 80 families of mothers, fathers, and their preschool-aged child (3 to 6 years) in two groups. The ASD group included 40 preschoolers (5 females reflecting the typical gender distribution in ASD) diagnosed with ASD by trained clinicians according to DSM-IV-TR criteria [44] and their parents. Families of children with ASD were recruited from psychiatric clinics and special-needs kindergartens and families of TD children were recruited through kindergartens and by ads posted in the community and on-line. Family demographics were matched for parental age and education (Table 1) and all children were raised in two-parent families who reported being of middle-class SES based on income level. All parents had no known psychiatric disorder and were physically healthy. Diagnosis was confirmed using the second edition of the Autism Diagnostic Observation Schedule (ADOS-2) [45], with 56% given module 2 and 44% module 3. Children in the ASD group underwent an extensive clinical diagnosis by a clinical psychologist and none met criteria for another psychiatric disorder in addition to ASD. The TD group included 40 preschoolers (6 females) and their parents with no neuro-psychiatric disorders who matched the ASD group on mental age, gender, and family demographics. TD participants were screened for ASD using the Childhood Autism Spectrum Test (CAST) [46]. Groups were matched on raw scores of Stanford-Binnet Intelligence Test [47]. Five children in the ASD group scored one or less SD from the average (Table 1). The study was approved by the Institutional Review Board of the Meir Medical Center, Kfar Saba, Israel, and all parents signed an informed consent.

**Table 1:** **Demographic information**

	**Total sample (** ***N*** **= 80)**	**ASD group**	**TD group**	***t*** **(78)**
	**Mean (SD) range**	**Mean (SD) range**	**Mean (SD) range**	
Child measures: age (months)	58.47 (13.93) 29 to 82	63.38 (12.35) 36 to 82	53.56 (13.83)29 to 78	3.31*
Verbal reasoning	14.83 (5.13) 1 to 43	14.15 (4.08) 7 to 21	15.51 (5.98) 1 to 43	1.17
Abstract/visual reasoning	13.36 (10.27) 1 to 80	12.67 (6.66) 3 to 27	14.05 (12.98) 1 to 54	0.59
Quantitative reasoning	11.35 (7.27) 1 to 54	11.15 (5.59) 1 to 20	11.54 (8.7) 1 to 80	0.23
Short-term memory	12.49 (6.26) 1 to 42	13.18 (4.86) 4 to 22	11.79 (7.58) 1 to 42	0.96
ADOS-2		11.89 (3.23) 7 to 22	N/S	
Demographic (years)				
Mother age	36.88 (4.45) 27 to 47	37.6 (4.45) 30 to 47	36.14 (4.39) 27 to 44	1.37
Father age	39.49 (5.14) 28 to 53	40.34 (5.33) 31 to 53	38.6 (4.86) 28 to 52	1.12
Mother education	16.26 (2.38) 12 to 25	15.94 (2.47) 12 to 22	16.59 (2.28) 12 to 25	1.42
Father education	16.39 (3.34) 12 to 28	15.97 (3.71) 12 to 25	16.87 (2.85) 12 to 28	1.11

*P < .05.

### Procedure

#### Participants

Children were visited in the kindergarten by trained psychologists for cognitive testing (all children) and clinical diagnosis (ASD group). In the ASD group, diagnosis was conducted using the second edition of the ADOS 2 [45], with 56% given module 2 of the ADOS and 44% module 3. One child failed to meet the ASD criteria and was excluded from the study. The TD group consisted of 40 preschoolers (6 females) and their parents, with no known neurodevelopmental or psychiatric diagnoses, who were matched to the ASD group on mental age, gender, and family demographics. TD participants were screened for ASD using the CAST [46]. To provide matching between groups on mental age, children in the TD group were slightly younger than children in the ASD group and groups were matched on raw scores of four subtests from the Stanford-Binet Intelligence Test [47] (Table 1) and the decision to match children on mental age was consistent with prior research [48,49]. Families received $80 in vouchers for their participation.

Two identical home visits were conducted within the same month, one with mother and one with father, and the order of the visit was counterbalanced. After a brief period of acquaintance, a baseline salivary sample was collected from parent and child. Following, the FTFSF was the first interactive paradigm in the visit. The FTFSF was followed by play with toys, a second CT sample from parent and child for reactivity, and several ER procedures: puppets - in which parent and child played with hand puppets, bubbles - in which the experimenter blew soap bubbles for parent and child to play, and masks, in which the experimenter wore four different masks and emotional reaction of child and parent are recorded. The social battery lasted approximately 45 min. Ten minutes after the completion of the social battery, the third CT sample for recovery was collected from parent and child.

##### The preschooler face-to-face still-face paradigms

In this age-modified version of the FTFSF paradigm [11], parent and child engaged in a 7-min free play with age-appropriate toys. Following, the parent was asked to maintain a ‘still-face’ for 3 min and then to resume play for additional 2 min. Parents were informed about the procedure as follows: ‘After a few minutes of playing together, you will hear a tap and you should stop playing and talking with your child and maintain a still face. If the child calls for attention, you should ignore or say you are busy now and cannot play. Three minutes later you will hear the tap again, this time to resume typical play and communication’. In the few cases parents responded during the still-face episode, they were gently reminded by the experimenter to resume the still face for a little longer and in most (>95%) cases parents were able to maintain the still face for the full 3-min period.

##### Cortisol

During both home visits, a baseline saliva sample was collected from parent and child after a brief period of acquaintance with the experimenter. The parent was asked to place a Salivette (Sarstedt, Rommelsdorft, Germany) in their own and the child’s mouth for 1 min. Visits were timed to the afternoon hours so that baseline cortisol was measured in all participants at 5 PM on a school day. All children attended school on the day of the home visit, woke up at approximately 7 AM, returned from school at 3 to 4 PM, and did not nap in the afternoon to avoid circadian changes in cortisol levels. Two additional cortisol (CT) samples were collected: CT *reactivity* was collected 10 min after the end of FTFSF episode (15 min from the initiation of the SF episode of the procedure) and CT *recovery* was collected 10 min after the end of the entire social battery. After the social battery, children remained in room for 10 min while their parents completed questionnaires until the last CT sample was collected. During this time, children did not engage in any effortful activity and typically relaxed or played with the toys. The timing of reactivity and recovery was based on our prior research on CT in high-risk preschoolers and their parents [50]. Of the 80 participants and 160 home visits, 6 children with ASD and 1 TD child refused to place the Salivate in their mouth, and CT for these children were not measured. In eight home visits, CT was not analyzed due to technical problems (for example, insufficient saliva).

#### Coding

Coding was conducted for each episode separately for each parent and child. Two sets of codes were applied: social behavior coded separately for the free play (FP), still-face (SF), and reunion (RE) episodes to assess SF effect, and regulatory behavior coded for the SF and RE episodes to examine regulatory behavior during social rupture and repair. Parent regulation-facilitation tactics were coded only for RE and addressed how the parent assisted the child in overcoming moments of social rupture. In Additional file 1 (word file), we provided a detailed description of each code. Codes are based on prior research on social synchrony, the FTFSF paradigm, and ER in preschoolers with ASD [11,17,51,52]. Coding was conducted by two graduate students in psychology on a computerized system (The Observer, Noldus Co., Wageningen, the Netherlands). The following variables were coded independently for each parent and child and percentages of time each behavior occurred out of the entire episode were measured.

##### Parent and child’s social behavior

The following social behaviors were coded; Negative emotionality/anger, withdrawal/sadness, positive vocalizations/laughter, and social gaze. These behaviors were coded three times (for FP, SF, and RE) for the child during each visit and twice (FP, RE) for each parent.

#### Child regulatory behavior

Two types of regulatory behaviors were coded consistent with prior research.

##### Putative regulatory behavior

This category included behaviors which are aimed solely for self-regulation and included physical self-sooth (for example, thumb-sucking), verbal self-sooth (for example, ‘that’s OK’), repetitive self-talk (‘don’t worry, don’t worry’), proximity seeking (child approaching parent), and idiosyncratic behavior (for example, hand flapping).

##### Complex regulatory behavior

This category included behaviors which are not inherently self-regulatory but may be used for ER during moments of increased stress and included substitutive-symbolic play (‘dolly’s hungry’), functional play (for example, moving a toy train back-and-forth), talking to parent, and re-orienting attention.

#### Parent regulation-facilitation

Two types of parental regulation-facilitation strategies were coded;

Simple regulation-facilitation - included behaviors parents typically employ with infants or toddlers and are mainly physical in nature [53], including providing physical comfort to child, touching/hugging, or simple attention diversion (look at this train!).

Complex regulation-facilitation - included behaviors that emerge during the preschool years and assist parents in facilitating child ER at this age [54], such as emotion regulation, such as cognitive reframing and emotional reflection (for example, ‘grandpa must have been so happy to see you’).

Inter-rater reliability was conducted for 20% of the interactions. Coders were first trained to 85% reliability (agreements/agreements + disagreements) on all codes. Following, reliability kappas were computed and kappa averaged = .86 (range = .80 to .95).

#### Cortisol

Salivettes were kept cooled until thawed before being centrifuged at 4°C at 1,000 × *g* for 15 min. The samples were then stored at −20°C until assayed. Cortisol levels were assayed using a commercial ELISA kit (Assay Design, Ann Arbor, MI, USA). Measurements were performed in duplicates according to the kit’s instructions and consistent with our prior research [51,55]. CT levels were calculated by MatLab-7 according to relevant standard curves. The intra-assay and inter-assay coefficients are <10.5% and 13.4%.

## Results

Results are reported in four sections. In the first, we report differences related to parent (mother, father) and group (ASD, TD) in the effects of social rupture (SF) on child and parent’s social behavior. In the second, we test differences in regulatory behavior of children and parents. The third section presents data on mothers’, fathers’, and children’s cortisol reactivity. Finally, Pearson’s correlations assess the inter-relatedness between child factors (symptom severity, IQ, baseline CT) and parent and child’s regulatory behavior. Prior to data analyses, we examined child gender effects on all study variables and none was found, and thus, analyses are reported across gender.

### Still-face effect on parent and child’s social behavior

#### Child social behavior

**Figure 1:**
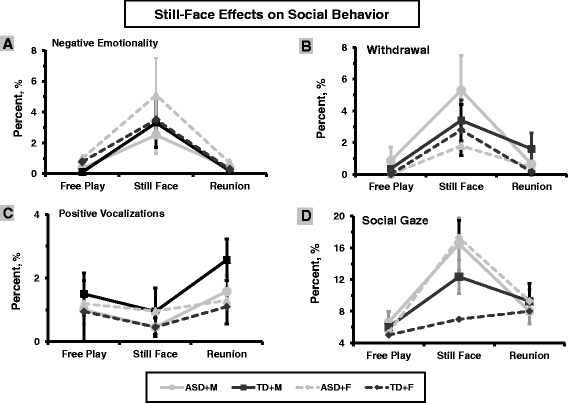
**Still face effects on social behavior in typically developing children and children with autism spectrum disorder during interactions with mother and father.** Four child social behaviors: **(A)** negative emotionality, **(B)** withdrawal, **(C)** positive vocalization, and **(D)** social gaze. Footer: TD + M, typically developing children with mother. ASD + M, children with autism spectrum disorder with mother. TD + F, typically developing children with father. ASD + F, children with autism spectrum disorder with father.

Four repeated-measure ANOVAs were computed to assess change in each social behavior (negative emotionality, withdrawal, positive vocalizations/laughter, and social gaze) as a function of episode (FP, SF, RE) and parent (mother, father) with group as the between-subject factor. Findings for the four social behaviors according to parent and group are presented in Figure 1A,B,C,D.

### Negative emotionality

An overall effect was found for episode: *F*(4,74) = 3.66, *P* = .009, effect size (ES - partial Eta squared) = .16, which was significant during interactions with both mother, *F*(1,74) = 4.272 = .016, ES = .053, and father, *F*(1,304) = 9.13, *P* < .001, ES = .10. Children expressed more negative emotionality/anger during the SF episode compared to FP, *t*(1,78) = 2.65, 2.87, *P* < .01, for mother and father respectively, and negative emotionality returned to baseline at RE. No group or group-by-episode interactions were found, indicating a similar pattern in TD and ASD.

### Withdrawal

Similar findings emerged for withdrawal. Significant overall effects were found for episode: *F*(4,73) = 4.009, *P* < .01, ES = .05: mother, *F*(4,74) = 6.05, *P* = .003, ES = .07; father: *F*(4,74) = 5.68, *P* = .004, ES = .07, with no group or interaction effect. Children expressed more withdrawal during the SF episode compared to FP, *t*(1,78) = 2.81, 2.87, *P* < .01, for mother and father respectively, and returned to baseline at RE with both parents.

### Positive vocalizations/laughter

Significant effect for episode emerged only during interaction with mother; *F*(2, 76) = 3.53, *P* = .034, ES = .04. Positive vocalizations/laughter was low during FP and SF, but both TD and ASD children increased positive vocalizations when mother resumed play after rupture, *t*(1,78) = 2.03, *P* < .05. No effects were found for father.

### Social gaze

An overall effect was found for episode: *F*(4,74) = 8.83, *P* < .001, ES = .32: mother, *F*(1, 74) = 40.27, *P* < .001, ES = .34; father: *F*(4,74) = 28.18, *P* < .001, ES = .27, indicating that child’s social gaze changed across the three episodes. A significant group-by-episode effect for fathers was found; *F*(4,74) = 5.14, *P* = .001, ES = .22. During mother-child FP, children mainly engaged in joint attention to toys, but gaze to the unresponsive mother increased during SF, *t*(1,78) = 4.15, and during RE children maintained high vigilance and social gaze did not return to baseline and was higher than during FP, *t*(1,78) = −1.98, *P* = .05. For fathers, the SF effect was found only in the ASD group. Among TD children, no differences were found between social gaze to father during FP, SF, and RE; however, for the ASD group, social gaze to the unresponsive father increased from FP to the SF episode, *t*(1,38) = 3.11, *P* = .004, and remained high at RE. During the SF episode, children with ASD showed more social gaze to their unresponsive parents compared to TD children *t*(1,78) = 1.99, *P* = .05.

### Parent social behavior

Parent’s social behavior (negative emotionality/anger, withdrawal, social gaze, positive affect/laughter) was compared for the two episodes where the parent participated actively - free play and reunion - and change in the parent’s social behavior from before to after social rupture was tested using repeated-measure ANOVAs (FP, RE) with group as the between-subject factor.

#### Mothers’ social behavior before (FP) and after (RE) social rupture

No episode or group effects emerged for negative-angry and withdrawn affect. However, for *positive affect*, there were both effects for episode, *F*(2,75) = 26.23, *P* < .001, ES = .22, and a group-by-episode interaction, *F*(2,75) = 51.11, *P* < .001, ES = .40, emerged. Mothers of TD children expressed no positive affect/laughter during FP and play was mainly characterized by mother and child playing with toys and expressing neutral affect rather than high positive arousal. After social rupture, mothers increased the expression of positive affect (M = 8.20%, SD = 10) possibly to restore social communication. On the other hand, mothers of children with ASD expressed higher levels of positive affect initially (M = 4.11%, SD = 5.1) and those remained unchanged at reunion (M = 5.32%, SD = 6.62), indicating that mothers of ASD children color their typical interactions with more positive arousal and focus on maintaining sameness of communication.

For *social gaze*, significant effects of episode, *F*(1,77) = 14.94, *P* < .001, ES = .16, and group, *F*(1,77) = 4.67, *P* < .05, ES = .04, were found. All mothers increased social gaze after rupture at reunion. However, mothers of children with ASD showed significantly more social gaze during both FP (TD: M = 15.87%, SD = 17.31; ASD = 22.64%, SD = 21.96) and RE (TD: M = 22.83%, SD = 23.16; ASD = 33.52%, SD = 27.02). These findings demonstrate the great effort mothers of children with ASD recruit in order to provide positive social environment and maintain a person-focus, particularly after rupture, possibly sensing the child’s difficulty in handling moments of maternal unavailability.

#### Fathers’ social behavior before (FP) and after (RE) social rupture

No effect emerged for fathers’ negative and withdrawn affect. With regard to *positive affect*, fathers in both groups expressed more positive affect after social rupture at reunion, *F*(1, 76) = 26.67, *P* < .001, ES = .26. During free play, no positive affect was expressed; however, during reunion, all fathers increased the expression of positive affect to resume play after social rupture (M = 5.43%, SD = .4.56).

Fathers’ *social gaze* showed a significant episode effect, *F*(1, 76) = 13.38, *P* < .001, ES = .14. Fathers in both groups expressed more social gaze at reunion (M = 23.41%, SD = 18.22) compared to FP (M = 16.23%, SD = 18.07). These data suggest that both mothers and fathers of TD and ASD children increase social involvement following rupture in order to resume positive communication.

#### Regulatory behavior during still-face and reunion in TD and ASD children

**Figure 2:**
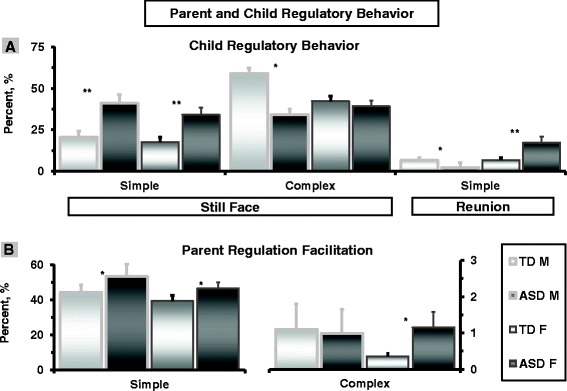
**Child regulatory behavior and parent regulation facilitation in typically developing children and children with autism spectrum disorder during interactions with mother and father. (A)** Child regulatory behavior. **(B)** Parent regulation-facilitation. Footer: *P < .05. **P < .01.

Child regulatory behaviors were tested during (SF) and following (RE) social rupture with repeated-measure ANOVA with group as between-subject factor. Differences in complex regulatory behaviors were measured only during SF, as it is difficult to judge whether behaviors which are not inherently regulatory and are expressed in non-stressful contexts (for example, symbolic play) serve a regulatory function. Child and parent’s regulatory behaviors are presented in Figure 2.

### Child regulatory behavior

#### Putative regulatory strategies

##### Child with mother

Significant effect emerged for episode, *F*(1, 76) = 38. 26.67, *P* < .001, ES = .33, and group, *F*(1, 76) = 6.48, *P* = .013, ES = .07. All children decreased their putative regulatory behaviors from SF to RE. Children with ASD used such behaviors significantly more during the SF episode compared to TD children, *F*(1,77) = 4.44, *P* < .05, but not during RE.

##### Child with father

Similar effect emerged for episode, *F*(1,76) = 12.37, *P* < .001, ES = .14, and group, *F*(1, 76) = 5.76, *P* = .019, ES = .07. Children decreased the use of putative regulatory behavior from SF to RE, but children with ASD used putative regulatory behavior significantly more during both SF and RE: *F*(1,77) = 9.73, *P* < .01 (Figure 2A).

#### Complex regulatory strategies

ANOVA assessing children’s use of complex regulatory strategies during the SF episode showed a parent by group interaction, *F*(1, 76) = 4.21, *P* = .041, ES = .05. During maternal SF, TD children employed more complex regulatory behavior than children with ASD, F = 5.63, *P* = .026; but no differences were found during paternal SF (Figure 2A).

#### Parent regulation-facilitation following social rupture

##### Simple regulation-facilitation

Repeated measure ANOVA assessing maternal and paternal simple regulation-facilitation strategies at RE showed an overall effect for parent, *F*(1,76) = 7.38, *P* = .008, ES = .09, and group *F*(1,76) = 4.45, *P* = .042, ES = .05. Mothers used more simple regulatory behaviors than fathers and more simple strategies were used by parents of ASD children as compared to parents of TD children.

#### Complex regulation-facilitation

A similar repeated-measure ANOVA showed a parent-by-group interaction effect, *F*(1,76) = 5.13, *P* = .026, ES = .03. This effect indicated that whereas no group differences were found between mothers of TD and ASD children in use of complex regulation-facilitation strategies, fathers of ASD children used more complex regulation-facilitation tactics than fathers of TD children (Figure 2B).

#### Cortisol reactivity in TD and ASD children with mother and father

##### Child and mother

**Figure 3:**
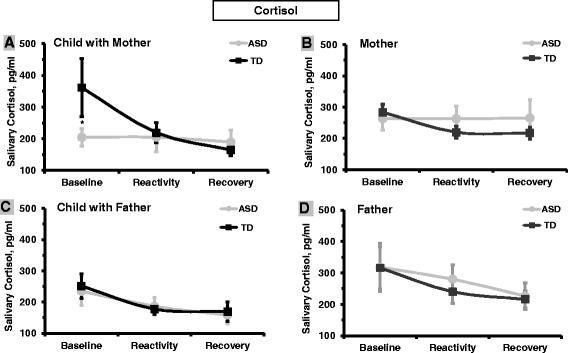
**Cortisol at baseline, reactivity, and recovery in mothers and fathers and in typically developing children and children with autism spectrum disorder during sessions with mother and father. (A)** Child with mother. **(B)** Mother. **(C)** Child with father. **(D)** Father. Footer: *P < .05.

Repeated-measure ANOVA of the three child cortisol assessments with mother revealed a main effect for assessment, *F*(2, 75) = 3.63, *P* = .031, ES = .14, indicating that cortisol changed over time, and an assessment-by-group interaction, *F*(2,75) = 3.42, *P* = .38, ES = .12, demonstrating that change over time differed among groups. Among TD children, CT was high at baseline and declined over time. On the other hand, children with ASD did not show the initial stress response when mother was present and no difference was found in CT levels between the three assessments (Figure 3A). Mothers’ cortisol showed decline over time, *F*(2, 75) = 4.33, *P* = .032, ES = .11, with no group or interaction effect (Figure 3B).

##### Child with father

Repeated-measure ANOVA of child cortisol with father, showed decline over time from initial high levels, *F*(2,73) = 10.10, *P* < .001, ES = .29, for both groups with no group or interaction effect (Figure 3C). Fathers’ cortisol similarly showed decline over assessment, *F*(2,73) = 6.67, *P* = .002, ES = .19, with no group or interaction effects (Figure 3D). Fathers’ CT also declined over time *F*(2, 75) = 4.65, *P* = .027, ES = .13, with no group or interaction effect. No differences were found between maternal and paternal CT. These findings may suggest that the human stress response reacts to social novelty (strangers entering the house with covered boxes), which declines over time, and that by 3 years of age TD children present the adult profile.

##### Correlations between parent and child’s cortisol

Medium to high correlations (*r* = .35 to .91) were found between each individual’s three cortisol assessments. Child baseline cortisol with mother and father showed cross-time stability, *r* = .43, *P* < .001. At each cortisol assessment (baseline, reactivity, recover), there were significant correlations between parent and child’s cortisol levels, with magnitude of correlations ranging from, *r* = .36, *P* < .01 to *r* = .62, *P* < .001. These correlations point to ‘endocrine fit’ [56] or ‘biological synchrony’ [57] between parent and child’s hormonal levels.

#### Correlations between child factors and parent and child’s social and regulatory behavior

Pearson’s correlations between child factors - including symptom severity on the ADOS, IQ, and baseline CT with mother and father - with parent and child’s regulatory behavior appear in Additional file 2. As seen, child symptom severity score correlated with lower IQ. Children with more severe ASD symptoms and with lower IQ tended to use more simple and less complex ER behavior during both SF and RE with mother and father. Child IQ and symptom severity were generally not related to measures of parent regulation-facilitation, apart from a negative correlation between child IQ and maternal simple strategies. Baseline cortisol with mother and father showed medium-level correlation but CT was unrelated to parent or child’s regulatory behavior. Child simple strategies showed significant correlations between the SF and RE episodes with mother and father, between SF with both parents, but not between RE with the two parents. Child complex strategies showed no stability between episodes or parents, indicating that the use of complex strategies may be more context-bound. The use of simple and complex strategies mainly showed negative correlations, and this probably resulted from our coding scheme that defined each regulatory behavior as either simple or complex. Finally, mothers’ and fathers’ simple and complex regulation-facilitation strategies were unrelated.

## Discussion

Results of the current study - the first to test social-regulatory and neuroendocrine patterns in preschoolers with ASD during interactions with mother and father - describe the behavioral response of preschoolers to moments of parental availability, the parent’s regulation-facilitation tactics following social rupture, and the neuroendocrine stress response of TD and ASD children to this social setting. Overall, our findings provide compelling evidence for the abilities of children with ASD to form social expectations and repair moments of social rupture. We found that children with ASD and TD children show remarkably similar social-emotional response to moments of maternal and paternal unavailability. All children exhibited increase in negative affect and withdrawal during the SF, which was repaired at reunion. Similarly, all children showed decline in positive affect during SF with mother, not with father, possibly owing to the more object-focused play of fathers [58]. Thus, despite measurable difficulties in reading facial expressions and understanding complex social situations [59], children with ASD appear to be as sensitive as their peers to parental disengagement, indicating that such moments violate their expectations of the parental ongoing support and attesting to the internalization of social rules of conduct during interaction with their parents. Furthermore, children in both groups increased their attempts to establish eye contact with the unavailable parent, pointing to their sense of social agency. Such findings are not trivial in the case of young children with ASD in light of their well-known impairments in initiating and maintaining joint attention and mutual gaze [60], and underscore the importance of the parent-child context for such children. Our findings are consistent with attachment research, similarly measured on the basis of children’s response to parental absence and return, which found the same prevalence of secure attachment in children with high functioning ASD to sensitive mothers. Thus, results of the FTFSF and strange situation paradigms converge in suggesting that young children with ASD form internalization of the parental ongoing support, the secure-base from which social-emotional growth emanates.

On the other hand, with regard to the child’s use of ER behavior, we found differences between TD and ASD preschoolers. Children with ASD showed more simple and less complex ER strategies during both maternal and paternal SF. Children’s ER capacities develop with age, and by the preschool years, children already possess a broad repertoire for regulating specific emotions [61]. Whereas infants use simple self-soothing behavior, including thumb sucking or simple disengagement tactics such as gaze aversion, toddlers and preschoolers can use more sophisticated strategies, such as exploratory play, symbolization, or focused attention, to regulate negative emotions [11]. With the development of increasingly sophisticated regulatory skills, children learn to regulate their emotions and actions more effectively in everyday contexts [62]. Our findings indicate that children with ASD use strategies typical of younger ages and are less inclined to engage in age-appropriate tactics and these findings are consistent with studies of ER behavior in preschoolers with ASD in child-alone situations [17]. One possible interpretation is that moments of parental unavailability are more anxiety-provoking to children with ASD and they are less able to divert from the unavailable parent, as seen by the finding that during the SF episode children with ASD looked more at the unresponsive parent. Such hypothesis is consistent with our finding that although children with ASD used more putative regulatory behavior during the SF episode, such behaviors dramatically decreased when the parent returned. During reunion, there were no group differences in the use of simple strategies, possibly due to the great scaffolding efforts parents of children with ASD use to help their children regulate stress [63].

Mothers and fathers of both TD children and children with ASD were well-aware of the child’s distress during social rupture and employed high levels (>40% of time) of simple regulation-facilitation tactics at reunion, including physical proximity, touch, and attempts to divert the child’s attention from the previous rupture. Parents of children with ASD were particularly sensitive to their child’s difficulties and employed even greater amounts of such simple, physical, and immediate regulation-facilitation behaviors. In addition, the use of complex strategies involving cognitive reframing and emotional reflection was employed by mothers and fathers of children with ASD to a greater extent than fathers (not mothers) of TD children. These data underscore the great effort both mothers and fathers of children with ASD recruit to sensitively buffer their children’s social stress, regulating social rupture through both elevated levels of simple strategies that are typically observed in younger children alongside comparable (to moms) and higher (than dads) levels of complex strategies. An alternative explanation to the increased use of simple strategies by parents of children with ASD may relate to the Broad Autism Phenotype theory [64]. According to this perspective, relatives of children with ASD, even those without a formal diagnosis, may share some traits of the autism phenotype. One such characteristic may be difficulty in using complex ER behavior and a tendency to use simpler strategies, and this may be observed in both parent and child. In general, our findings are consistent with research showing increased regulation-facilitation effort among mothers of children with ASD [22] and the use of simple, immediate strategies to achieve regulatory goals [17]. Since ours is among the first studies to compare fathers’ regulation-facilitation behavior in TD and ASD children, the findings that fathers of children with ASD showed high levels of both simple and complex regulation-facilitation tactics, which were comparable to those of mothers, highlight their sensitivity to the child’s ongoing regulatory needs and emphasize the importance of including fathers in intervention efforts in this population [65].

Results regarding the child’s neuroendocrine patterns revealed an intriguing picture. Among TD children with mother and father and children with ASD with father, an initial stress response to social novelty was observed, which was down-regulated as the visit progressed. It appears that at this age the FTFSF in of itself does not elicit an HPA stress response, and possibly, after the first months of life the stress system learns to adjust to momentary parental ‘failures’ and can auto-regulate such daily social stress. However, among children with ASD during interaction with mother, no initial stress response was observed and cortisol levels were blunted throughout the visit. It has been shown that following an early sensitive period when neonatal rodents’ HPA response is blunted, there is a transitional period when the typical HPA response exists but is suppressed by maternal presence. This stage gives way to the mature profile when mothers no longer regulate infant CORT, enabling transition to the outside world and response to its dangers. Our findings similarly show that the typical cortisol response in children with ASD exists (with father) but is suppressed by maternal presence, as observed during the transition period before the mature self-regulated HPA function develops in rodents. These findings may suggest that one mechanism in the pathophysiology of ASD may relate to abnormal extension of the HPA sensitive period, with mothers exerting social buffering effect for much longer periods and the system not maturing to its full adult profile for extended intervals. This may point to the involvement of GABAergic processes, which control the initiation and termination of critical periods in ASD but this hypothesis requires much further research.

Importantly, several behavioral findings similarly point to a more ‘typical’ response with father and a more ASD-specific profile with mother. For instance, patterns of social gaze and positive affect during the FP, SF, and RE showed no group differences with father and more group differences with mother. In addition, children with ASD displayed less complex regulatory strategies during mother-child sessions but exhibited comparable levels to those of TD children during sessions with father. Finally, fathers of children with ASD used more complex regulation facilitation tactics than fathers of TD children. Possibly, fathers demand more from their ASD child compared to mothers and in the father’s presence children function at a higher developmental level. These findings again emphasize the need to further understand the effects of supportive fathering in this population and underscore the importance of including fathers in intervention efforts for children with ASD.

The lack of change in ASD children’s cortisol response during mother-child sessions parallels the behavioral results for mothers’ positive affect. Whereas mothers of TD children expressed little positive affect during free play and increased positive expressions after rupture, mothers of children with ASD engaged in higher levels of positive affect initially and maintained similar levels of positive emotionality at reunion, anticipating both their child’s difficulty in regulating maternal disengagement and their need for sameness in emotional atmosphere. These findings highlight the great investment and burden placed on mothers of children with ASD, who must provide external-regulatory support to their child’s emotion-regulatory and physiological stress response long after the typical sensitive period by means of careful online adaptation to social cues and the maintenance of sameness in emotionality. These findings are also the first, to our knowledge, to demonstrate that similar assessment with mother and father revealed a different profile; one similar to TD, the other unique to ASD. Such findings emphasize the importance of testing physiological and behavioral outcomes in this population with both parents, as assessment with mother only would not have revealed that children with ASD can exhibit the typical neuroendocrine repertoire.

Biological synchrony - correlations between parent’s and child’s cortisol response - was found in TD and ASD children with both mothers and fathers. These findings demonstrate, for the first time, biological synchrony between cortisol levels in children with ASD with both parents and is consistent with our bio-behavioral synchrony model [57,66], which describes the online co-regulation of social and physiological processes during moments of social contact within affiliative bonds. These results demonstrate that the HPA system of children with ASD is open to the ongoing influences of the attachment figure, similar to that observed in prior research for TD infants [28] and children [45,53]. The inter-correlation matrix shows associations between the child’s symptom severity and less mature ER tactics, that is, with more simple and less complex ER behavior during both SF and RE with mother and father. These findings indicate that although ER difficulties are not considered a diagnostic criterion for ASD, difficulties in ER are highly prevalent in this group and ER problems increase in more symptomatic children. These findings also lend support to the current study paradigm and coding scheme and demonstrate that our measures capture a meaningful aspect of the child’s psychopathology and its severity.

## Conclusions

Limitations of the study primarily relate to the lack of longitudinal data, which could have shed further light on how individual differences in children’s response to social rupture and repair shape their later social-regulatory competencies. It is also important to remember that cortisol levels show wide individual variability and results regarding CT should be interpreted with caution. Assessing other neuroendocrine systems and brain patterns could have contributed to a more comprehensive understanding. Our findings have important implications for intervention by highlighting the role of fathers in ASD and underscore the importance of constructing interventions that target ER skills and utilize the parent-child context to enhance regulatory capacities. Such interventions may help children with ASD acquire more complex, language-based mechanisms to regulate moments of social distress, and enable them to transfer the online biological and behavioral synchrony formed with mother and father to other members of their social world.

## Additional files

Additional file 1:
**Word file detailing the coding scheme.** Coding of child emotional reactivity and emotion regulation and parent regulation facilitation behaviors during the free play, still face and reunion paradigms. Additional file 2: Table S1. Correlations among child factor and child and parent’s regulatory behavior.

## Abbreviations

ADOS: Autism Diagnostic Observation Schedule
ANOVA: analysis of variance
ASD: autism spectrum disorder
CAST: Childhood Autism Spectrum Test
CT: cortisol
ER: emotion regulation
FP: free play
FTFSF: face-to-face-still-face (paradigm) including three parts
HPA: hypothalamic-pituitary-adrenal (axis)
MANOVA: multivariate analysis of variance
RE: reunion
SF: still face
TD: typically developing (children)
