# Supplementary material for: Neuroendocrine and behavioral response to social rupture and repair in preschoolers with autism spectrum disorders interacting with mother and father
Source: Mol Autism. 2015 Mar 6;6:11. doi: 10.1186/s13229-015-0007-2 (PMC4359452; doi:10.1186/s13229-015-0007-2)
Supplement: Additional file 1: — Word file detailing the coding scheme. Coding of child emotional reactivity and emotion regulation and parent regulation facilitation behaviors during the free play, still face and reunion paradigms. [file 13229_2015_7_MOESM1_ESM.docx]

Coding of Child Emotional Reactivity and Emotion Regulation and Parent Regulation Facilitation Behaviors during the free play, still face and reunion Paradigms

|  | **Emotionality (parent and child)** |
| --- | --- |
| Facial expressions of fear, or sadness, such as brow pulled down, lips pulled down, etc. sometimes accompanied by body expressions of sadness such as sloping shoulders, vocal tone sad or quiet, or crying.  Facial expressions of anger, or frowning, such as brow pulled down, lips chuckled , etc. sometimes accompanied by body expressions of anger such as foot stomping etc., vocal town angry, or shouting.  Facial expressions expressing joy, exuberance, or happiness, such as lips pulled up and eyes narrowed (smiling), sometimes accompanied by body expressions of enthusiasm, such as jumping, clapping hands, or pulling up arms. Child often smiles or laughs.  Facial expressions neutral or relaxed. Such as no muscular tension on facial muscles etc. vocal tone neutral with regular peach. | Negative withdrawn affect  Negative angry affect  Positive affect  Neutral affect |
|  |  |
| Behaviors aimed at avoiding the target object after it has been presented, such as hiding face or whole body, turning or twisting body from object, moving backward from target object, walking away, or escaping the room.  Repetitive unusual behaviors with no apparent goal. These may include hand flapping, body rocking, finger flips, head movement, tongue clicking, lips smacking, etc.  Bodily-directed behaviors aim to self-sooth, such as thumb sucking, hair-twisting, self-petting, laying down etc.  Turning focus away from the target object or partner to active play with another object. | **Child's Regulatory behavior strategy**  Withdrawal  Idiosyncratic behaviors  Physical self-soothing  Solitary substitutive play |
| Child is looking for physical closeness with parent, approaching parent, cuddling, putting head on parent's lap, taking parent's hand  Child makes verbal request for parent to help or play, or actually plays with parent with toys or physical mutual play.  Child actively resists the interaction, throws a tamper tantrum. Or tries to hit the parent.  Child talks without communicative intent. includes verbal self soothing, private speech during pretend or functional play and cognitive reappraisal.  Child talks to parent concerning the interaction, or during mutual play including also requests and comments.  Child talks to experimentor includes questions, requests resistence and attempts to involve experimentor in play.  Child repeats same word or phrase more than twice.  Parent initiates physical contact to provide comfort or sooth child, including physical proximity, hugging, patting, giving hand etc.  Parent employs child's attention by presenting toys, exclaiming, or suggesting an alternative game .or parent engaged in mutual play.  Parent seems uninvolved in the interaction, passive or engaged in non relevant activities (answering the phone)  Parents talks to child, hums, or sings in order to sooth and provide comfort.  Parent reflects or elaborates on the child's emotional state (e.g "Oh! this is a scary lion, is it?", "are you afraid?", "you're laughing, it’s funny"  Parent attempts to regulate the child's emotional state by reframing the situation or the experienced emotion (e.g "do you remember we saw the same lion in the zoo. It was so much bigger")  Parent distracts child's attention by talking about other topics unrelated to the task (e.g. "How was your day"),  Parent initiates mutual communication through play, babbling, conversations, vocalizations or responding to the child's play in words. | Proximity seeking  Playing with parent  Defiance  **Child's regulatory verbal strategy**  self talk,  talk to parent ,  talk to experimenter,    repetitive talk  **Parent's Regulatory behavior strategy**  Physical comfort/touch  Diverting or playing  Withdrawal  **Parent's Regulatory verbal strategy**  Verbal comfort  Emotional reflection  Cognitive reappraisals  Diverting/Disracting attention  Interactive communication |
|  |  |
